# Quantitative PCR used to Assess HIV-1 Integration and 2-LTR Circle Formation in Human Macrophages, Peripheral Blood Lymphocytes and a CD4+ Cell Line

**DOI:** 10.1186/1743-422X-7-354

**Published:** 2010-12-03

**Authors:** Brian Friedrich, Guangyu Li, Natallia Dziuba, Monique R Ferguson

**Affiliations:** 1 Department of Internal Medicine, Division of Infectious Diseases, University of Texas Medical Branch, Galveston, Texas 77555-0435, USA

## Abstract

**Background:**

Integration is an intermediate step in the HIV life cycle and is defined as the insertion of HIV-1 proviral DNA into the host chromosome. If integration does not occur when HIV-1 cDNA enters the nucleus, it circularizes upon itself and forms a 2-LTR circle. Monitoring the level of integrated HIV-1 cDNA in different primary cell subsets is very important, particularly regarding the effect of HAART in HIV-1 infected individuals. Because of limitations of prior HIV-1 integration assays, there is limited data on the level of integration and 2-LTR circle formation in primary cell subsets, particularly in human monocyte-derived macrophages and peripheral blood lymphocytes (PBL).

**Results:**

In this study, we utilized a well-defined, sensitive two-step quantitative real-time PCR method to detect HIV-1 integration as well as conventional real-time PCR to detect 2-LTR circle formation in human macrophages and PBL isolated from six different healthy donors, as well as U373 CD4^+ ^cells by infecting with HIV-1_SX _(R5) or dual-tropic isolate HIV-1_89.6 _(R5/X4) virus strains. We used the FDA-approved integrase inhibitor, raltegravir, to determine quantitative differences of integrated HIV viral cDNA in HIV-1 infected cells with and without raltegravir treatment. Our results show that integration and 2-LTR circle formation can be assessed in primary macrophages, PBL, and a CD4+ cell line by this method. Specifically, our results demonstrate that this two-step real-time PCR method can distinguish between HIV-1 integrated viral cDNA and non-integrated nuclear HIV-1 2-LTR circles caused by impaired integration with raltegravir-treatment. This further confirms that only integrated HIV-1 cDNA can be specifically amplified and quantified by two-step PCR without non-specifically detecting non-integrated viral cDNA.

**Conclusion:**

These results consistently demonstrate that the well-established real-time PCR assays used are robust, sensitive and quantitative for the detection of HIV-1 integration and 2-LTR circle formation in physiologically relevant human macrophages and PBL using lab-adapted virus strains, instead of pseudovirus. With two-step real-time PCR, we show that unintegrated, nuclear HIV-1 cDNA is not detected in raltegravir-treated cells, while specific for only integrated HIV-1 cDNA in non-treated cells. These methods could be applied as a useful tool in further monitoring specific therapy in HIV-1 infected individuals.

## Background

Human immunodeficiency virus type 1 (HIV-1) is known to infect several primary cell types, predominantly CD4^+ ^T lymphocytes and macrophages. HIV-1 infection results in a gradual decline in the number of CD4^+ ^T cells, leading to the development of AIDS. Macrophages are also of particular importance for the pathogenesis of HIV-1, as the cells are likely to be the major cell type involved in mucosal transmission of HIV-1 [1-3]. In addition, macrophages appear to be more resistant to the cytopathic effects of HIV-1 infection, so they are thought to play a crucial role in viral persistence, latency, and dissemination [4,5].

Early steps of HIV-1 infection include viral entry by binding to the main receptor CD4 and either of two co-receptors CCR5 or CXCR4. Upon membrane fusion, the viral core is released into the cytoplasm. Once inside the cell, reverse transcriptase converts viral RNA into DNA which is then transported into the nucleus and integrates into the host chromosome. Integration, the intermediate step of the HIV-1 lifecycle, is dependent on viral integrase activity for efficiently spreading infection [6-10]. If HIV-1 cDNA enters the nucleus but does not integrate into the host cell chromosome, then the viral cDNA circularizes to form a 2-LTR circle [11,12]. Advent of more sensitive assays for HIV-1 integration can enhance our knowledge of how cellular factors play a role in HIV-1 integration [13,14]. Previous methods to quantify integrated viral DNA include one-step amplification [15], nested linker primer PCR (LP-PCR) [16], virus-specific primer with tag sequence [17], and real-time nested PCR using *Alu*-specific primers [18,19].

Liszewski *et al. *described the limitations of each assay and recently showed that this two-step *Alu-gag *PCR method has high sensitivity as well as robust quantitation [18]. Since this two-step *Alu*-*gag *PCR assay is well-defined and highly sensitive and specific, we used this assay for detecting and quantifying integration in our cell subsets. Additionally, while the previous studies utilized pseudotyped virus in their assays, we used clinical, lab-adapted HIV-1 strains to measure the level of integrated DNA in human macrophages, peripheral blood lymphocytes (PBL) and U373 CD4^+ ^cell lines. We also employed the use of the FDA-approved integrase inhibitor, raltegravir. Because raltegravir prevents HIV-1 integration and causes formation of HIV-1 2-LTR circles, this allowed us to quantitatively assess the differences between integrated HIV-1 proviral DNA and unintegrated HIV-1 cDNA in HIV-1 infected cells.

## Results

### HIV-1 Integration in U373 cells

**Figure 1:**
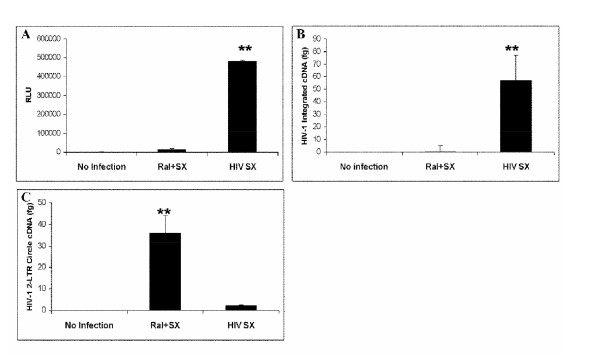
**Quantitation of HIV-1 integration and 2-LTR circle formation in CD4+ U373 cells**. U373-MAGI-CCR5 cells were plated in 6-well plates with or without raltegravir treatment 24 h prior to infection and during infection (MOI = 0.1). Two days after infection, (**A**) β-galactosidase activity (expressed as RLU = Relative Light Units) was analyzed for determination of HIV-1_SX _infection; (**B**) cellular genomic DNA was extracted from U373 cells 48 h after infection and HIV-1_SX _integration was detected using two-step quantitative PCR, and (**C**) 2-LTR circle formation was measured by real-time PCR. (**p < 0.01)

To verify *Alu*-*gag *two-step PCR could be used to detect HIV-1 integration system, HIV-1_SX_, a CCR5-tropic virus strain, was used to infect U373-MAGI-CCR5 cells (MOI = 0.1) with or without raltegravir treatment (Merck & CO. Inc., Whitehouse Station, NJ). FDA-approved raltegravir blocks HIV-1 integration by preventing strand transfer, and thus preventing HIV-1 from successfully inserting its viral cDNA into the host chromosome [20,21]. Forty-eight hours post-infection, cellular genomic DNA was isolated from U373 cells for detection of HIV-1 integration; meanwhile, β-galactosidase activity was analyzed for determination of HIV-1 infection. As shown in Figure 1A β-galactosidase activity in raltegravir treated cells with HIV-1_SX _infection was not seen, similar to control condition (non-infected/non-treated cells). However, there was a 6-fold increase in HIV-1_SX _infected cells without raltegravir treatment. In Figure 1B, HIV-1 integration was shown to be significantly different between infected cells with and without raltegravir treatment (P < 0.01), indicating detection of integration in HIV-infected cells in the absence of raltegravir treatment. Figure 1C shows that raltegravir-treated cells prevent integration and is the only treatment causing formation of 2LTR circles. This also confirms specificity for HIV-1 integration because non-integrated HIV-1 cDNA is not amplified by these real-time PCR probes. Three independent experiments were performed, and the data were consistent each time, proving to be a reproducible and reliable method for detection of integration in U373 cells.

### HIV-1 Integration in human PBL

**Figure 2:**
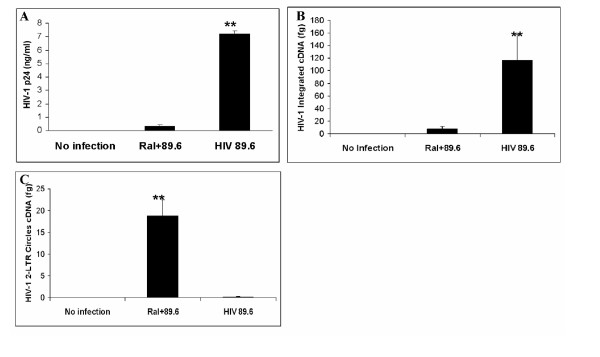
**Quantitation of HIV-1 integration and 2-LTR circle formation in human PBL**. PBL were plated in 6-well plates with or without raltegravir treatment 24 h prior to infection with HIV-1_89.6_, during infection and 48 h after infection (MOI = 0.1). (**A**) Seven days after infection, supernatant was assessed for p24 level of each group by p24 capture ELISA; (**B**) Six days after infection, cellular genomic DNA was extracted from PBLs and HIV-1 integration was measured by two-step quantitative PCR, and (**C**) 2-LTR circle formation was measured by real-time PCR. (**p < 0.01)

In order to assess integration in primary cell subsets, PBL were isolated from human blood, and infected with dual-tropic virus strain HIV-1_89.6_. As shown in Figure 2A, virus production (HIV-1 p24 measured by ELISA) in infected PBL was significantly lower (more than 7-fold) with raltegravir treatment compared to those without raltegravir (P < 0.01). The integration data (Figure 2B) was highly consistent with p24 data, showing HIV-1_89.6 _integration as significantly higher (more than 6-fold) in infected cells without raltegravir compared to raltegravir-treated cells (P < 0.01). In addition, Figure 2C shows that raltegravir treatment does increase 2LTR circle formation. These data are representative of six experiments in PBL.

### HIV-1 Integration in human macrophages

**Figure 3:**
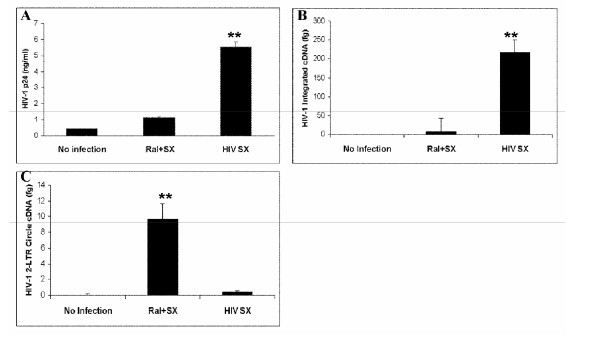
**Quantitation of HIV-1 integration and 2-LTR circle formation in human macrophages**. Macrophages were plated in 6-well plates with or without raltegravir treatment 24 h prior to infection, during infection and 48 h after infection (MOI = 0.1). (**A**) Seven days after infection, supernatant was assessed for p24 level of each group by p24 capture ELISA; (**B**) Six days after infection, cellular genomic DNA was extracted from macrophages, and HIV-1_SX _integration was measured by two-step quantitative PCR, and (**C**) 2-LTR circle formation was measured by real-time PCR. (**p < 0.01)

Human monocyte-derived macrophages were isolated from human blood, and infected with HIV-1_SX_. As shown in Figure 3A, virus production in infected macrophages was approximately 5-fold higher in cells without raltegravir treatment as compared to those with raltegravir treatment (P < 0.01). Similarly to other cell types, macrophages treated with raltegravir show a significant decrease in viral cDNA integration into the genome when compared with the cells without raltegravir treatment (P < 0.01), as shown in Figure 3B. Figure 3C shows that raltegravir-treatment increases 2LTR circle formation. For all cell systems used in this study, there was no cytotoxicity observed in raltegravir-treated cells (data not shown).

Taken together, these results suggest that this two-step quantitative PCR method can be used effectively to quantitate HIV-1 integration in primary human macrophages and PBL, as well as our CD4^+ ^U373 cell line.

## Conclusions

We used the antiretroviral integrase inhibitor, raltegravir, to distinguish between integrated and non-integrated HIV-1 cDNA in infected primary PBL, macrophages, and a human CD4^+ ^cell line. We detected HIV-1 integration by utilizing a well-defined two-step quantitative PCR method [19], which has proven to be a specific and sensitive approach in different cell subsets based on our reproducible results. In both raltegravir-treated and non-treated cells, viral RNA is reverse transcribed into viral cDNA and transported into the nucleus. In non-treated cells, viral cDNA integrates into the host chromosome, as detected by two-step real-time PCR; whereas in raltegravir-treated cells, viral cDNA forms 2-LTR circles preventing it from integrating into the host chromosome, as shown by conventional real-time PCR. Yu *et al. *have used this method to show that patients on HAART have decreased levels of integrated HIV-1 proviral DNA as compared to patients off HAART [22]. Thus, this method may be considered for the routine analysis of HIV-1 DNA integration to evaluate the integration efficiency of retroviral vectors in different cell subsets.

Our study extends the previous work performed by others [18,19] to detect integration in primary human cell subsets - PBL and macrophages using this two-step PCR technique. This is important because macrophages and PBL are crucial for HIV-1 infection, latency, and persistence [4,5]. As such, we infect human macrophages or PBLs derived from six different healthy donors, as well as infect a CD4+ cell line, and consistently demonstrate similar results using two different virus strains. By using these primary cell subsets, we show that this method can be useful in precisely monitoring the level of integration in laboratory settings and perhaps in HIV-infected patients to conclusively determine if it is affected by specific antiretroviral therapy. Thus, by using raltegravir as a control, we demonstrate that two-step PCR is specific in detecting only integrated HIV-1 cDNA and not other HIV-1 cDNA in the nucleus or cell. Additionally, we utilized lab-adapted R5- and dual-tropic strains of HIV-1 instead of pseudovirus to more closely mimic natural infection. Furthermore, this approach could reveal if HIV-1 integration persists within specific cellular subsets in patients on highly active antiretroviral therapy (HAART).

## Methods

### U373 cells

U373-MAGI-CCR5 cells (contributed by Drs. Michael Emerman and Adam Geballe), are modified U373 glioblastoma cells that are used for HIV infection experiments. U373-MAGI-CCR5 cells express β-galactosidase under the control of HIV LTR, which is trans-activated by HIV Tat protein in relation to the level of virus replication [23,24]. In addition, these cells express CD4 and human chemokine receptor CCR5 on its surface, which allow infection by primary HIV R5 strains [24]. U373 cells were propagated in 90% DMEM supplemented with 10% fetal bovine serum, 0.2 mg/ml G418, 0.1 mg/ml hygromycin B, and 1.0 μg/ml puromycin. For infection experiments, U373 cells were maintained in 90% DMEM, 10% fetal bovine serum, and 1% penicillin/streptomycin.

### Preparation of human PBL

PBL were isolated from PBMC obtained from six different healthy human buffy coats prepared by the University of Texas Medical Branch (UTMB) Blood Bank in Galveston, TX. After the initial 24 h incubation of PBMC on 10 cm petri dishes, supernatant (containing PBL) was transferred to 50 ml tube and cells were isolated by centrifugation. Cells then were resuspended in stimulation media (RPMI 1640 media with 20% Fetal calf serum (FCS); 1% Penicillin/Streptomycin; 5 μg/ml phytohemagglutinin) and incubated at 37°C with 5% CO_2 _for 72 h. PBL were then collected by centrifugation and resuspended in growth media (RPMI 1640 with 1% L-glutamine; 1% Penicillin/Streptomycin; 20% FCS; 20 units/ml IL-2).

### Preparation of human macrophages

Primary human macrophages were purified from healthy human PBMC (from the same six blood donors as human PBL isolation) by adherence to plastic tissue culture dishes as described previously [25]. Briefly, PBMC were purified by Ficoll-Hypaque centrifugation from buffy coats of healthy HIV-negative blood donors prepared by the UTMB Blood Bank. Primary monocyte-derived macrophages were obtained by adherence for 7 days to plastic petri dishes initially coated with human AB serum [26]. During differentiation, macrophages were cultured in Iscove's modified Dulbecco's medium supplemented with 20% FCS; 1% L-glutamine and 1% Penicillin/Streptomycin.

### Viruses and infection

HIV-1_SX_, which is a chimeric M-tropic virus (R5) encoding the majority of the HIV-1_JRFL _envelope protein in an HIV-1_NL4-3 _backbone, and dual-tropic (R5/X4) HIV-1_89.6_, which is a HIV-1 laboratory adapted strain originally isolated from infected individuals, were purchased from the Virology Core Facility, Center for AIDS Research at Baylor College of Medicine, Houston, TX. HIV-1_SX _stock containing 69.681 ng/ml of HIV p24 with 65,325 TCID50/ml was used to infect macrophages and U373 cells. HIV-1_89.6 _stock containing 49.977 ng/ml of HIV p24 with 261,300 TCID50/ml was used to infect PBL. HIV-1 stocks were titrated, and for all experiments, the inoculum was 7 ng of p24 per 1.5 × 10^5 ^cells (MOI 0.1). Raltegravir (Merck & Co., Inc., Whitehouse Station, NJ) is a well-characterized, FDA-approved HIV-1 integrase inhibitor. It had been previously tested in our lab and showed no visual cytopathic effects or any cytotoxicity at 20 μM (data not shown). U373-MAGI cells, primary macrophages, and PBL were plated in 6-well plates at 1.5 × 10^5 ^cells per well 24 h prior to infection. Each of these cell subsets was plated into three 6-well plates. In the first plate, cells were infected with HIV-1 only; the second plate was treated with raltegravir (20 μM) 24 h prior to HIV-1 infection and during infection; the third plate contained non-infected/non-treated cells serving as a negative control. After 4 h incubation of virus inoculum (0.5 ml/well) at 37°C, fresh medium (1.5 ml) was added to each well. For macrophages and PBL, genomic DNA was extracted 6 days post-infection using DNeasy Blood and Tissue Kit (QIAGEN, Alameda, CA) according to the manufacturer's instructions. To assess infection, supernatant was harvested for HIV p24 levels in each group by a p24 capture ELISA kit (Immuno Diagnostics, Inc, Woburn, MA) according to the manufacturer's instructions. Since the HIV replication kinetics are more rapid in U373-MAGI cells than in primary macrophages and PBL, genomic DNA was extracted from U373 cells 48 h post-infection. To assess infection of HIV-1SX in U373 cells, the cells were lysed and analyzed for β-galactosidase activity using the Beta-Glo Assay System (Promega, Madison, WI) and a Dynex MLX Luminometer.

### PCR

For the pre-amplification of genomic DNA from macrophages, PBL, and U373 cells the following primers were used: *Alu *forward, 5'-GCC TCC CAA AGT GCT GGG ATT ACA G-3'; and HIV-1 *gag *reverse, 5'-GCT CTC GCA CCC ATC TCT CTC C-3' [18,19]. The PCR solution contained 1× TaqMan Universal Master Mix, No AmpErase UNG (Applied Biosystems, Carlsbad, CA), 100 nM *Alu *forward primer, and 600 nM *gag *reverse primer, and 5 μl of DNA for every 15 μl of PCR solution. The Thermocycler (Applied Biosystems GeneAmp PCR system 2700) was programmed to perform a 2 min hot start at 94°C, followed by 30 steps of denaturation at 93°C for 30 seconds, annealing at 50°C for 1 minute, and extension at 70°C for 1 minute 40 seconds.

### Quantitative real-time PCR

For quantitation of HIV-1 integration, a second round real-time quantitative PCR was performed using 7 μl of the material from the pre-amplification step. These samples were run along with known dilutions of HIV-1_SX _plasmid cDNA used for a standard curve. This standard curve was used to quantify the amplified DNA. The sequences of the primers used are as follows: *LTR *forward, 5'-GCC TCA ATA AAG CTT GCC TTG A-3'; and *LTR *reverse, 5'-TCC ACA CTG ACT AAA AGG GTC TGA-3' [19]. The *LTR *molecular beacon probe, labeled on the 5' terminus with the reporter fluorophore 6-carboxyfluorescein (FAM) and on its 3' terminus with Black Hole Quencher 1 (DBH1), had the following sequence: 5'-FAM-GCG AGT GCC CGT CTG TTG TGT GAC TCT GGT AAC TAG CTC GC-DBH1-3' [19]. For quantitation of HIV-1 2-LTR circles, small non-genomic DNA was isolated from cells using a Qiagen Miniprep kit. To identify 2-LTR circle formation, primers MH535 (5'-AAC TAG GGA ACC CAC TGC TTA AG-3') and MH536 (5'-TCC ACA GAT CAA GGA TAT CTT GTC-3') were used with the MH603 probe (5'-(FAM)-ACA CTA CTT GAA GCA CTC AAG GCA AGC TTT-(TAMRA)-3') [27]. All reactions were performed in a volume of 20 μl containing 1× TaqMan Universal Master Mix, No AmpErase UNG, and 200 nM of forward primer, reverse primer, and molecular probe. All reactions were performed using Applied Biosystems TaqMan Universal Master Mix and run using an Applied Biosystems 7500 Fast Real-time PCR system and 7500 Fast System Software. The thermal program started with 2 min at 50°C, followed by a 10 minute hot start at 95°C. This was followed by 40 cycles of 95°C for 15 seconds and 60°C for 60 seconds. GAPDH was used as an internal control to normalize total DNA.

### Statistical analysis

To evaluate the sensitivity and specificity of this method, we detected the quantity of integration in three different cells, and compared them by student's *t-*test to determine differences between raltegravir treated groups and virus only infection groups. P < 0.05 was considered as significant difference.

## Competing interests

The authors declare that they have no competing interests.

## Authors' contributions

BF and GL performed all experiments and drafted the manuscript. ND participated in the design of the study and contributed to drafting the manuscript. MRF conceived of the study, and participated in its design and coordination and helped to draft the manuscript. All authors read and approved the final manuscript.
